# Retrospective analysis of Desmoplastic Ameloblastoma: Clinical review

**DOI:** 10.4317/medoral.24152

**Published:** 2020-10-09

**Authors:** Ankit Sharma, Snehal Ingole, Mohan Deshpande, Deepashree Meshram

**Affiliations:** 1 MDS, Oral and Maxillofacial surgery, Assistant Professor. Department of Oral and Maxillofacial surgery, R R Dental College and Hospital Opposite Umra Railway Station, Umarda, Udaipur, Rajasthan; 2 MDS, Oral and Maxillofacial surgery, Additional Professor. Department of Oral and Maxillofacial surgery, Nair Hospital Dental College, Mumbai, Maharashtra, India; 3 MDS, Oral and Maxillofacial surgery, Assistant Professor, Department of Oral and Maxillofacial surgery, Nair Hospital Dental College, Mumbai, Maharashtra, India

## Abstract

**Background:**

Desmoplastic Ameloblastoma (DA) is a rare, true neoplasm of jaws with reported incidence of 4-13% among other variants of Ameloblastoma, however this appears distinct than the classic Ameloblastoma in anatomical distribution and clinical presentation. This is often mistaken as a fibro-osseous lesion because of its similar radiological appearance.

**Material and Methods:**

To describe the clinical, radiographic and histopathological characteristics through a series of new cases of histologically proven DA including a case of an exceptionally large, recurrent lesion along with retrospective analysis of cases from literature available for an improved understanding of the behaviour and prognosis of DA. A total of 50 cases were analysed for the anatomical distribution, radiographic presentation and management. Out of the 50 cases, 47 cases were from the English literature reported from 2011 to 2019 and 3 were new cases.

**Results:**

DA showed a slight male predilection (male: female=1.17:1) with a predominance in the fourth and fifth decade of life. Mandibular involvement (52%) was more commonly seen with a marked tendency for the anterior region. Radiographically, most of the lesions presented mixed radiopacity with radiolucency(80%) and root displacement was observed in only 70.27 % cases. Recurrence rate of 26 .47 % was observed. Cases treated with resection resulted in lesser recurrence as compared to those treated with enucleation and curettage.

**Conclusions:**

DA is distinguished by a peculiar display of clinicalopathological parameters. DA has tendency of local disposition and propensity of recurrence, which thus necessitates its aggressive management. It is not possible to conclude or report on the aggressive/recurrent nature and appropriate treatment modality for DA due to inadequate follow-up results.

** Key words:**Desmoplastic ameloblastoma, mixed radiopaque – radiolucent, odontogenic tumors, recurrence, enucleation, curettage, resection.

## Introduction

Ameloblastoma is recognized as a common odontogenic tumor that accounts for about 1% of all the cysts and tumors of jaws and 11-59% of the odontogenic tumors (1). Clinically, it presents in three forms: Solid or Multicystic (SMA), Unicystic, and Extraosseous. Various histological subtypes that have been described for this neoplasm; which include Follicular, Plexiform, Basal, Granular, Acanthomatous, and Desmoplastic (2). The reported incidence of Desmoplastic Ameloblastoma (DA) in the literature ranges from 4 to 13 % of all Ameloblastomas (3). Contrary to the posterior region of mandible being the most common anatomical location for SMA, DA involves the anterior-premolar region of jaws more frequently. Clinically DA appears as a slowly growing small tumor and seldom attains large size. The Radiographic appearance of DA is often mixed radiopaque radiolucent with ill-defined borders unlike the more typical soap bubble or honeycomb appearance of SMA. This often creates a deception of DA being a fibro-osseous lesion. The histopathology of DA is also distinct, characterized by marked desmoplasia of connective tissue stroma with irregular and squeezed islands of odontogenic epithelium without palisading of columnar cells may mimic histologically as odontogenic fibroma (4). Although there exist few differences, DA has some similarities with SMA in its clinical behaviour. The lesion has the ability to locally infiltrate, potential to grow to a large size and is susceptible to recur therefore it requires aggressive treatment similar to SMA. The World Health Organization (WHO) of 2005 classification of odontogenic tumors included DA as a separate clinical variant of Ameloblastoma (5). However, it was re-classified as one of the histological subtype of Ameloblastoma by WHO in 2017 (6). Given the fact that DA being a very rare entity and fewer cases have published in the medical literature, the true clinical and biological profile of DA is still not well known. The purpose of this article is to describe the peculiarities of DA through a case series of 3 histologically proven cases including a case of the exceptionally large lesion and to review the existing literature for a clearer understanding of its behaviour and prognosis.

## Material and Methods

An electronic search of papers written in English-language literature published in PubMed/ MEDLINE, Web of Science, Science Direct between 2011 and 2019 was undertaken.

- Inclusion Criteria

The conditions for including the cases as a Desmoplastic type of Ameloblastoma were:

1) Specific details about the age and gender of the patient;

2) An appropriate detailed radiographic image and definition in the report or copies of the radiographs; and

3) Histological diagnosis.

Since this was a retrospective study, ethical approval was not required from Institutional Ethical committee. The Helsinki Declaration guidelines were followed. Informed consent was obtained from the patients reported in case series. Based on above inclusion criteria, the literature search resulted in 19 articles with 47 cases of DA. (4,7-24) Table 1 shows a comparison of the demographic, clinical and radiographic features of DA.

- Data analysis

The data was collected from the published literature. A total of 50 cases of DA, 47 cases reported in literature and 3 cases in the present case series were analysed. A descriptive analysis was performed based on mean ± standard deviation (SD) and percentage values in Microsoft Excel version 10 for windows. Analysis of the Data obtained is summarized in Table 2.

- Case Series

**Figure 1:**
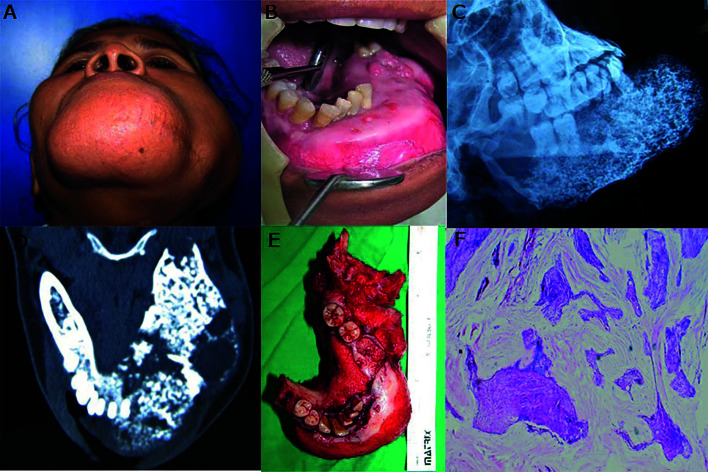
A-F, Large recurrent swelling in left mandible crossing midline (A and B), Plain Radiograph and axial CT shows ill-defined, expansile, mixed radiopaque radiolucent lesion (C and D), resected lesion (E), microscopic features of DA (F).

Case 1- A 50-year-old female patient reported with a complaint of longstanding large swelling on the left side of the face since 3 years. The patient was operated 2 years ago for the same; details of surgical procedure were unknown. The swelling had increased in size gradually and had also caused loosening and avulsion of teeth. The swelling was asymptomatic but caused discomfort due to its massive size and facial disFigurement. On clinical examination, there was gross asymmetry of the face due to large swelling extending from left mastoid posteriorly and crossing the midline of mandible anteriorly. On palpation, the swelling was warm, hard, and non-tender. Lips were incompetent. A scar of previous surgery was seen on the inferior border of the mandible (Fig. 1). Intra-orally swelling involved the entire left mandible crossed the midline and extended up to right canine region with the expansion of buccal and lingual cortices.

**Table 1:**
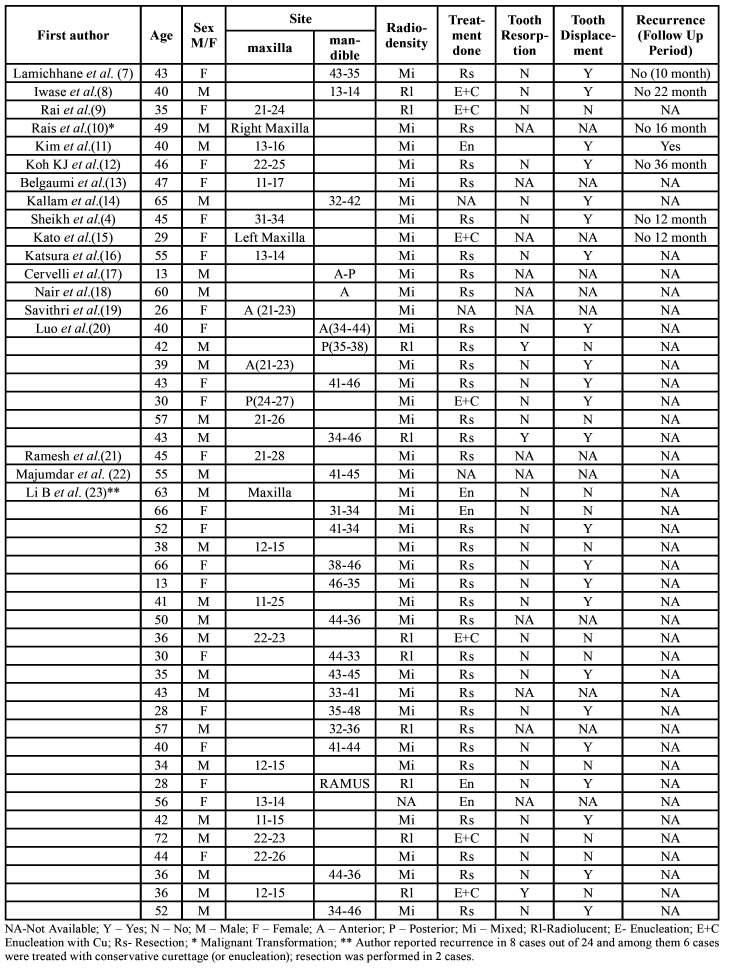
Distribution of Clinical and Radiological data of Desmoplastic Ameloblastomas.

**Table 2:**
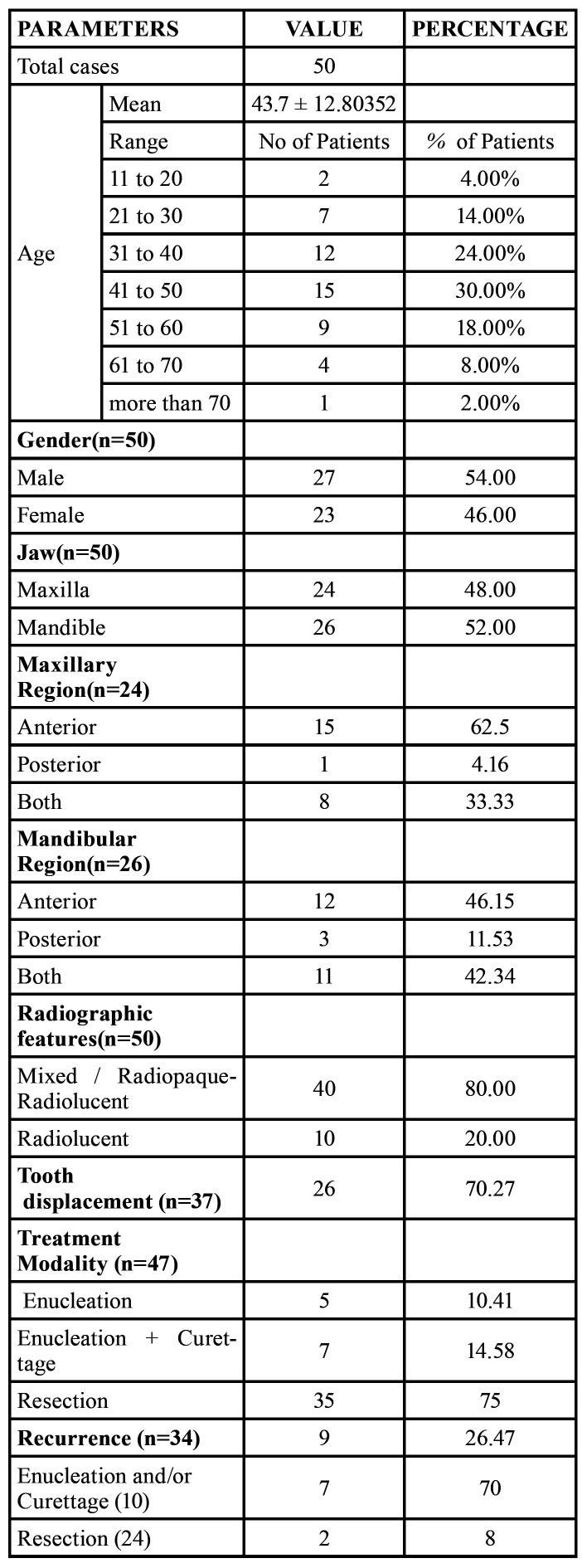
Summary of clinicopathological data for desmoplastic ameloblastomas.

The canine, premolars, and first molar on the left side were missing and remaining teeth involved in the swelling were mobile. Ulcerations were seen in anterior and posterior regions of the mandible which may be attributed to trauma from upper teeth. Blood vessels appeared stretched and compressed over the lesion (Fig. 1). Lesion appeared as large ill-defined radiopaque radiolucent lesion involving the left hemi-mandible on plain radiograph (Fig. 1). CT scan revealed a large expansile heterogenous lesion extending from right first molar to left ramus of the mandible. The lesion appeared as a mixed radiolucent radiopaque structure with ill-defined borders resembling a fibro-osseous lesion (Fig. 1). All laboratory investigations were carried out and found to be within normal limits. Also, the calcium, phosphorus, and alkaline phosphatase concentrations were within normal limits. The tumor was pathologically diagnosed as ameloblastoma on incisional biopsy. Surgical resection with disarticulation of left mandible and primary reconstruction with a titanium plate was done. The resected mass measured about 11cm x 6cm x 7cm in size, firm in consistency, non-capsulated, irregular in shape (Fig. 1). Histopathological examination of the resected specimen confirmed it as Desmoplastic Ameloblastoma (Fig. 1). The patient was kept on regular follow up and no signs of recurrence were seen after 5 years of follow up.

**Figure 2:**
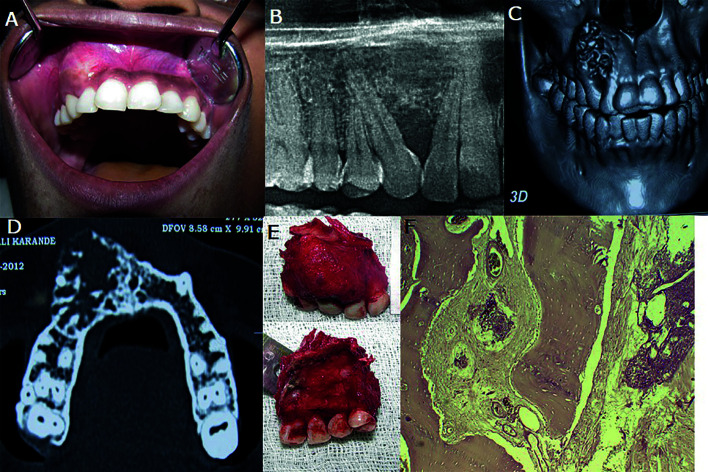
A-F, Swelling in right maxilla (A), OPG and CT shows ill-defined, expansile, radiopaque-radiolucent lesion extending from alveolar region to pyriform rim with root displacement (B,C and D), partial maxillectomy (E), histopathological features of DA (F).

Case 2- A 30 year old female patient reported with a complaint of a slow-growing painless swelling in the right anterior maxilla. A well-defined, ovoid 2x2cm, hard and non-tender swelling was seen over the right supralabial region with mild obliteration of right nasolabial fold. Intraorally, the swelling involved the right lateral incisor, canine and first premolar, extending into and obliterating right labial vestibule. The overlying mucosa appeared normal. The involved teeth were immobile (Fig. 2). On the Panoramic radiograph, the lesion appeared ill-defined mixed radiopaque radiolucent with the displacement of lateral incisor and canine (Fig. 2). 3DCT examination showed the lesion extending from alveolar process of maxilla to pyriform rim on the right side. (Fig. 2). The lesion appeared as a mixed radio-opaque radiolucent, expansile lytic lesion in the right maxilla with buccal cortical plate expansion and perforation involving right anterior and premolar teeth (Fig. 2). Hematological findings were within normal limits. Incisional Biopsy was suggestive of ameloblastoma. Partial maxillectomy of right maxilla with the extraction of left central incisor, right incisors, canine, and premolars was done (Fig. 2). Microscopically, the lesion appeared as desmoplastic ameloblastoma (Fig. 2). The postoperative course was uneventful; prosthetic rehabilitation for the missing teeth was done. The patient was under regular follow up and showed no signs of recurrence after 4 years.

**Figure 3:**
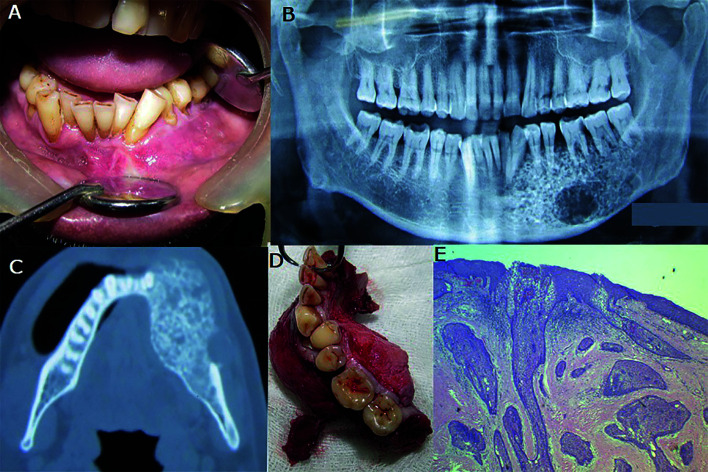
A-E, Swelling in left body of mandible (A), OPG shows ill-defined, mixed radiopaque radiolucent lesion (B), Axial CT shows ill-defined, expansile lesion in left mandible (C), Segmental resection of mandible (D), microscopic appearance of DA (E).

Case 3- A 60-year-old male patient reported with a complaint of an asymptomatic swelling in the posterior left mandible for three and a half months. On clinical examination, a hard non-tender swelling approximately 6cm x 3cm was present in the left body of the mandible. The skin over the swelling appeared normal. Left submandibular lymph nodes were palpable and non-tender. Intraoral examination revealed a well-defined, hard, non-tender swelling extending from left central incisor to left molar region obliterating labial and left buccal vestibule of the mandible (Fig. 3). Radiograph showed an osteolytic lesion with multiple discrete radiopacities extending from left lateral incisor to the left third molar of the mandible. Expansion and perforation of buccal and lingual cortical plates were seen in the left second premolar and molar region (Fig. 3). CT scan revealed an expansile lesion involving left body and angle mandible measuring 6cm x 3.3cm x 3.2cm with mixed radiolucent radioopaque appearance (Fig. 3). The left inferior alveolar canal was not traceable. Incisional Biopsy of the lesion was suggestive of ameloblastoma. Segmental resection with a wide margin of left body mandible was done and the defect was reconstructed with a titanium plate (Fig. 3). Histologically, the features of the resected specimen were consistent with those of desmoplastic ameloblastoma (Fig. 3). 4-year follow up of the patient did not show any signs of recurrence.

The clinical and radiographic findings of the three cases are summarized in (Table 3).

**Table 3:**
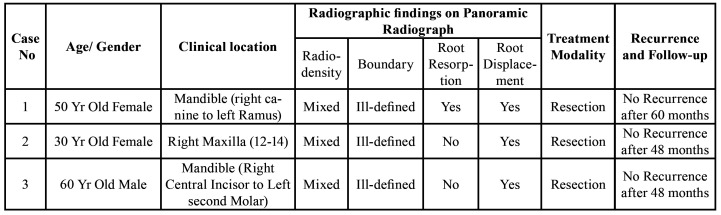
Summary of the three cases of Desmoplastic Ameloblastoma.

## Results

A total of 50 histologically proven cases of DA were analysed. 54 % of patients affected with DA were male and 46% were female. DA in this study was seen to affect patients’ age ranging from 13 to 72 years with a mean age of 43.7 ± 12.80.

48 % of cases were in maxilla and 52% cases involved mandible. DA was seen to involve both anterior and posterior regions of jaws. On the radiograph, 80 % cases of DA had a mixed radiopaque-radiolucent appearance while 20 % of cases appeared radiolucent. Root displacement of involved teeth was noted in 70.27 % of cases.10.41 % of cases were treated by enucleation, and curettage with enucleation was done in 14.58 % cases of DA. Resection with or without continuity defect was adopted in 75% cases of DA.

## Discussion

Desmoplastic ameloblastoma (DA) is a true jaw neoplasm arising from the odontogenic epithelium and has been recognized as one of the unusual variants of ameloblastoma with 4-13 % recorded incidence among other variants of ameloblastoma (24). Philipsen *et al*. in their review of 100 cases reported a higher incidence of DA in Americans and Europeans while the Japanese were believed to be less affected (25). In contrast, higher incidence in Asia with maximum cases among Japanese were observed by Sun *et al*. in a retrospective analysis of 115 cases of DA in the literature (3). However, further rigorous analysis is required to determine the true geographic distribution of DA. Our experience with the incidence of DA is 3 out of 71 histologically proven cases of ameloblastoma in the last 8 years which makes about 4.23 % of total cases of Ameloblastoma.

DA is more likely to impact people in the 4th and 5th decades. The mean age of 43.7 ± 12.80 was observed in this study, which is slightly higher compared to the age of 42.9 years reported by Philipsen *et al*. (25) Similar observation was reported in a systematic review of 238 reported cases by Anand *et al*. (26) , where 31.72 % patients were between 41 to 50 year age. About 15-17 % of patients of classical ameloblastoma are below 20 years of age, (27) however, the exact incidence of DA is not known. In this review, we observed that 3.92% of patients were below 20 year age group. Mean age of patients reported in the case series is 46.66.

Males and females are reported to be equally affected. A ratio of 1.17:1 between males and females was found in our study, which is in accordance with the finding of Li *et al*. (23) Interestingly we in our review observed less number of males than females below 30 years of age. Though this finding can be coincidental; it indicates that females are affected at a younger age as compared to males. However, more rigorous and extensive analysis with the higher number of cases of DA is required to establish this fact.

Earlier reports have shown the same frequency of DA in both the maxilla and mandible relative to solid multicystic ameloblastomas affecting mandible more generally with a variable mandible maxilla ratio of 90:1 to 5:1 (28). In this study, the site was identified in 50 cases, 24 of which occurred in the maxilla (48%), and 26 (52%) in the mandible. Among the 24 cases in the maxilla, the anterior region displayed the most frequent involvement (15/24, 62.5%), followed by the anterior-posterior region (8/24, 33.33%) and (1/24, 4.16%) were limited to the posterior area. Similarly, for the mandible, the anterior region (12/26; 46.15%) was the most commonly affected area followed by the antero-posterior region (11/26; 42.34%) and the posterior region (3/26; 11.53%). Both the cases in mandible in the case series had involved both anterior and posterior region the case reported in maxilla was limited to anterior region. Such findings are of particular clinical importance and are decisive in determining the required intervention. The less compact maxilla and its proximity to the sinus would cause the tumor mass to penetrate rapidly, thereby requiring aggressive therapy and therefore presenting a challenge in terms of recurrence control and further rehabilitation of the defect after excision.

Clinically, DA presents itself as slow-growing swelling or tumor of the jaw mostly without pain or mild to moderate pain in some cases much like other ameloblastomas, which does not aid in the identification of this variant. Radiologically different presentations of the DA have been reported in the literature. The most common appearance is the mixed radio-opaque radiolucent with ill-defined boundaries and hence DA can be wrongly diagnosed as fibro-osseous lesions. Various explanations have been given for this characteristic appearance of DA. Philipsen *et al*. (29) proposed osteoplasia as a reason for mixed appearance. Takata *et al*. (30) believed the infiltrative behaviour of the lesion is responsible for the characteristic appearance of DA. Thompson *et al*. (31) correlated the mixed radio-opaque, radiolucent appearance, and ill-defined boundaries histologically with the bone remodelling in response to the expansion of lesion in bony trabeculae and the infiltration of the collagenous substance of lesion. Radiolucent appearance is reported in less number of cases of DA. In agreement with the previous reviews on radiographic appearance, this review also found the majority of the cases having mixed appearance (80 %, 40/50). Additionally, 70.27% of cases were associated with root displacement which is another significant radiographic finding. All three cases in this report had mixed radiographic appearance and ill-defined borders with expansion and perforation of cortical plates. Root displacement was present in all the cases.

**Figure 4:**
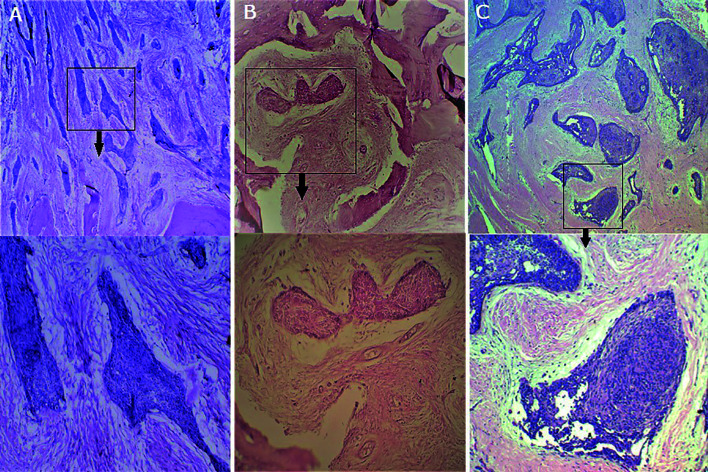
A-C, Histopathology of Desmoplastic Ameloblastoma in all 3 cases showing stretched-out irregular odontogenic islands in a dense desmoplastic stroma (above ), at higher magnification of same images (below) showing odontogenic islands having hypercellular spindle or polygonal cells in central part and squamous or flattened, cuboidal, cells at periphery.

DA is distinctive in its histological nature and is distinct from classical ameloblastoma as the palisaded peripheral columnar cells with reversed polarity and the inner stellate reticulum-like cells are not observed. The connective tissue stroma shows substantial collagenisation with few bony trabeculae and the odontogenic islands appear stretched or distorted and composed of flattened cuboidal or squamous cells at the periphery. Columnar cells with nuclear polarity are seldom visible (6). Three of our cases had histological features typical of DA (Fig. 4). Immuno-histochemically also DA exhibits striking differences from classical ameloblastoma. The stromal component of DA has been reported to show high reactivity to Type I and IV collagen and fibronectin as compared to SMA (32). Interpretation of DA biopsy specimens must be carried out with utmost caution because focal desmoplasia can also be found in SMA (33). Besides, the lesion may be falsely diagnosed as an odontogenic fibroma displaying epithelium and calcification. This differential diagnosis is important as it would have an impact on the choice of treatment as odontogenic fibromas are treated conservatively, while resection is typically used for DA (4,34).

 Various treatment modalities have been used in the treatment of DA depending upon size anatomic location and proximity to the vital structure. Most reports recommend resection as the preferred method of excision due to the infiltrative nature of the lesion. The most common surgical intervention followed in various cases in this review was resection (75%, 36/48) followed by Enucleation with Curettage (14.58%, 7/48) and least commonly Enucleation alone in (10.41% 5/48). Lack of documentation and long term follow-up precludes accurate assessment of best form of treatment and prognosis for this lesion it was observed that resection led to a lower risk of recurrence in comparison to enucleation and / or curettage in the treatment of DAs. Recurrence rate in the present review was 26 .47 %( 9/ 34) cases. The recurrence rate using conservative surgical therapy through enucleation and/or curettage is significantly higher 70% (7/10) compared to 8.34 % (2/24) post resection. These observations clearly demonstrate the inadequacy of enucleation and/or curettage. Resection yields good results, particularly if carried out including the wide margins. All three cases in this report were treated with resection and were followed up for 4 years, and none of the cases showed any signs of recurrence to date. Although this study did not compare ameloblastomas of different histological types, the observation of this study has a higher recurrence in DA as compared to other histopathological variants which is similar to the observation made by Keszler *et al*. (35).

## Conclusions

DA appears as a true neoplasm of odontogenic epithelium distinguished with other intraosseous variants of Ameloblastoma by a peculiar display of clinicopathological parameters. These lesions have a tendency of local disposition and propensity of recurrence, thus necessitates the aggressive management of Desmoplastic ameloblastoma. It is not possible to report on its aggressive/recurrent nature and appropriate treatment modality due to inadequate follow-up results. A lot more comprehensive DA reports are needed, including long-term follow-up.

## References

[1] Reichart PA, Philipsen HP, Sonner S (1995). Ameloblastoma: biological profile of 3677 cases. Eur J Cancer B Oral Oncol.

[2] Masthan KM, Anitha N, Krupaa J, Manikkam S (2015). Ameloblastoma. J Pharm Bioallied Sci.

[3] Sun ZJ, Wu YR, Cheng N, Zwahlen RA, Zhao YF (2009). Desmoplastic ameloblastoma - A review. Oral Oncol.

[4] Sheikh S, Pallagatti S, Singla I, Kalucha A (2011). Desmoplastic ameloblastoma: a case report. J Dent Res Dent Clin Dent Prospects.

[5] Fulco GM, Nonaka CF, Souza LB, Miguel MC, Pinto LP (2010). Solid ameloblastomas - Retrospective clinical and histopathologic study of 54 cases. Braz J Otorhinolaryngol.

[6] Sivapathasundharam B, Biswas PG, Preethi S (2019). The World Health Organization classification of odontogenic and maxillofacial bone tumors: An appraisal. J Oral Maxillofac Pathol.

[7] Sharma Lamichhane N, Liu Q, Sun H, Zhang W (2016). A case report on desmoplastic ameloblastoma of anterior mandible. BMC Res Notes.

[8] Iwase M, Fukuoka A, Tanaka Y, Saida N, Onaka E, Bando S (2017). Hybrid Desmoplastic/Follicular Ameloblastoma of the Mandible: A Case Report and Review of the Literature. Case Rep Pathol.

[9] Rai S, Misra D, Prabhat M, Jain A, Jain P (2019). Hybrid Ameloblastoma of Anterior Maxilla: A Rare and Puzzling Pathologic entity - Case Report with Systematic Review. Contemp Clin Dent.

[10] Rais R, El-Mofty SK (2019). Malignant Transformation of a Desmoplastic Ameloblastoma to Squamous Cell Carcinoma: A Case Report. Head Neck Pathol.

[11] Kim JD, Jang HS, Seo YS, Kim JS (2013). A repeatedly recurrent desmoplastic ameloblastoma after removal and allobone graft: Radiographic features compared with histological changes. Imaging Sci Dent.

[12] Koh KJ, Park HN, Kim KA (2015). Desmoplastic variant of ameloblastoma of the maxilla: A case report. Imaging Sci Dent.

[13] Belgaumi UI, Sundaresh KJ, Varma S, Mallikarjuna R (2013). Desmoplastic ameloblastoma: a rare odontogenic neoplasm with unusual radiographic and histomorphological presentation. BMJ Case Rep.

[14] Kallam SR, Arutla R, Gadwalwari SS, Kubbi JR, Shylaja SR (2015). Desmoplastic Ameloblastoma - An Unusual Presentation. J Clin Diagn Res.

[15] Kato H, Nomura J, Matsumura Y, Tagawa T (2011). A case of desmoplastic ameloblastoma occupying maxillary sinus. Contemp Clin Dent.

[16] Katsura K, Maruyama S, Suzuki M, Saku T, Takagi R, Hayashi T (2011). A case of desmoplastic ameloblastoma arising in the maxillary alveolus: the origin and time-course changes in the early stage of tumour development observed on dental radiographs. Dentomaxillofac Radiol.

[17] Cervelli D, Marianetti TM, Boniello R, Grussu F, Gasparini G, Azzuni C (2012). Giant neglected desmoplastic ameloblastoma: reconstruction with free fibula flap. J Craniofac Surg.

[18] Nair PP, Bhat GR, Neelakantan S, Chatterjee R (2013). Desmoplastic ameloblastoma of mandible. BMJ Case Rep.

[19] Savithri V, Janardhanan M, Suresh R, Kumar RV (2013). Desmoplastic ameloblastoma with osteoplasia: Review of literature with a case report. J Oral Maxillofac Pathol.

[20] Luo J, You M, Zheng G, Xu L (2014). Cone beam computed tomography signs of desmoplastic ameloblastoma: review of 7 cases. Oral Surg Oral Med Oral Pathol Oral Radiol.

[21] Ramesh V, Singh S, Bailwad S, Kiran K, Agarwal R, Singh A (2014). The complexity of stromal changes in desmoplastic ameloblastoma. Ann Med Health Sci Res.

[22] Majumdar S, Uppala D, Kotina S, Veera SK, Boddepalli R (2014). Desmoplastic ameloblastoma. Int J Appl Basic Med Res.

[23] Li B, Long X, Wang S, Cheng Y, Chen X (2011). Clinical and radiologic features of desmoplastic ameloblastoma. J Oral Maxillofac Surg.

[24] Hendra FN, Van Cann EM, Helder MN, Ruslin M, de Visscher JG, Forouzanfar T (2020). Global incidence and profile of ameloblastoma: A systematic review and meta-analysis. Oral Dis.

[25] Philipsen HP, Reichart PA, Takata T (2001). Desmoplastic ameloblastoma (including "hybrid" lesion of ameloblastoma). Biological profile based on 100 cases from the literature and own files. Oral Oncol.

[26] Anand R, Sarode GS, Sarode SC, Reddy M, Unadkat HV, Mushtaq S (2018). Clinicopathological characteristics of desmoplastic ameloblastoma: A systematic review. J Investig Clin Dent.

[27] Andrade NN, Shetye SP, Mhatre TS (2013). Trends in Pediatric Ameloblastoma and its Management: A 15 year Indian Experience. J Maxillofac Oral Surg.

[28] Filizzola AI, Bartholomeu-dos-Santos TC, Pires FR (2014). Ameloblastomas: clinicopathological features from 70 cases diagnosed in a single oral pathology service in an 8-year period. Med Oral Patol Oral Cir Bucal.

[29] Philipsen HP, Ormiston IW, Reichart PA (1992). The desmo- and osteoplastic ameloblastoma. Histologic variant or clinicopathologic entity? Case reports. Int J Oral Maxillofac Surg.

[30] Takata T, Miyauchi M, Ito H, Ogawa I, Kudo Y, Zhao M (1999). Clinical and histopathological analyses of desmoplastic ameloblastoma. Pathol Res Pract.

[31] Thompson IO, van Rensburg LJ, Phillips VM (1996). Desmoplastic ameloblastoma: correlative histopathology, radiology and CT-MR imaging. J Oral Pathol Med.

[32] dos Santos JN, De Souza VF, Azevêdo RA, Sarmento VA, Souza LB (2006). "Hybrid" lesion of desmoplastic and conventional ameloblastoma: immunohistochemical aspects [published correction appears in Rev Bras Otorrinolaringol (Engl Ed). 2006 Nov-Dec;72(6):852]. Braz J Otorhinolaryngol.

[33] Ngwenya SP, Raubenheimer EJ, Noffke CE (2009). Internal morphology of ameloblastomas: a study of 24 resected specimens. Oral Surg Oral Med Oral Pathol Oral Radiol Endod.

[34] Lam KY, Chan AC, Wu PC, Chau KY, Tideman H, Wei W (1998). Desmoplastic variant of ameloblastoma in Chinese patients. Br J Oral Maxillofac Surg.

[35] Keszler A, Paparella ML, Dominguez FV (1996). Desmoplastic and non-desmoplastic ameloblastoma: a comparative clinicopathological analysis. Oral Dis.

